# Rare Case of Budd-Chiari Syndrome in a Young Child: A Diagnostic Conundrum

**DOI:** 10.7759/cureus.16407

**Published:** 2021-07-15

**Authors:** Yumna Timsaal, Syed Hasan Ali, Farheen Malik, Ashok Chawla, Jawad Ahmed

**Affiliations:** 1 Internal Medicine, Dow University of Health Sciences, Karachi, PAK; 2 Pediatrics, Dr. Ruth K. M. Pfau Civil Hospital Karachi, Karachi, PAK

**Keywords:** budd-chiari syndrome, bcs, thrombosis, vascular liver disorder, portal hypertension, liver cirrhosis

## Abstract

Budd-Chiari syndrome (BCS) is an uncommon vascular disorder in which venous thrombosis prevents the venous outflow of the liver. The obstruction is primarily at the level of hepatic veins and inferior vena cava. Here, we present a case of a two-and-a-half-year-old male child who presented with complaints of abdominal distension for two months and fever and watery diarrhea for one month. Physical examination showed the patient was anemic with palmar erythema. He was started on an empirical treatment of cefotaxime, metronidazole, and amikacin. Sensitivity and culture reports for blood and urine samples were negative, but abdominal computed tomography (CT) scan showed characteristic findings for BCS with caudate lobe hypertrophy. After the symptomatic treatment of the patient, a liver transplant was suggested as a last resort.

## Introduction

Budd-Chiari syndrome (BCS) is a group of clinical conditions presenting with hepatic venous outflow obstruction from the level of the hepatic veins to the junction of the inferior vena cava (IVC) with the right atrium. The median age for disease occurrence is between the third and fifth decade of life [1]. Pediatric BCS is relatively rare, with an incidence of one per one million across the world annually [2]. To our knowledge, only two cases of BCS in children have been reported in Pakistan to date [3,4].

The classical triad of BCS includes hepatomegaly, ascites, and abdominal pain. Depending on the stage of the disease, BCS can be classified as acute or chronic. These two classifications differ in clinical presentations, treatment modalities, and prognostic factors [5]. The acute onset of BCS is characterized by hepatic dysfunction, abdominal pain, ascites, and, at times, renal failure [5,6]. Chronic presentation of the disease accompanied by portal hypertension is seen more often [6]. BCS is a rare cause of liver cirrhosis; it tends to be misdiagnosed and receives delayed treatment [7]. However, early diagnosis and clinical intervention are crucial to prevent BCS patients from developing life-threatening complications of liver failure and portal hypertension [8]. Here, we present a case of a two-and-a-half-year-old child who presented to the emergency department with complaints of fever, abdominal distention, and watery diarrhea and was diagnosed as a case of chronic BCS.

## Case presentation

A two-and-a-half-year-old male child, fully vaccinated up to his age, presented in the pediatric emergency department of Dr. Ruth K. M. Pfau Civil Hospital Karachi with complaints of progressive abdominal distension for two months accompanied by fever and watery diarrhea for one month. The stools were yellow, loose, and foul-smelling, occurring up to four to five times per day. History was unremarkable for blood in stool, vomiting, or alternating constipation. The patient was the second product of consanguineous marriage and delivered full-term via normal vaginal delivery. The patient reached developmental milestones at appropriate ages.

**Figure 1:**
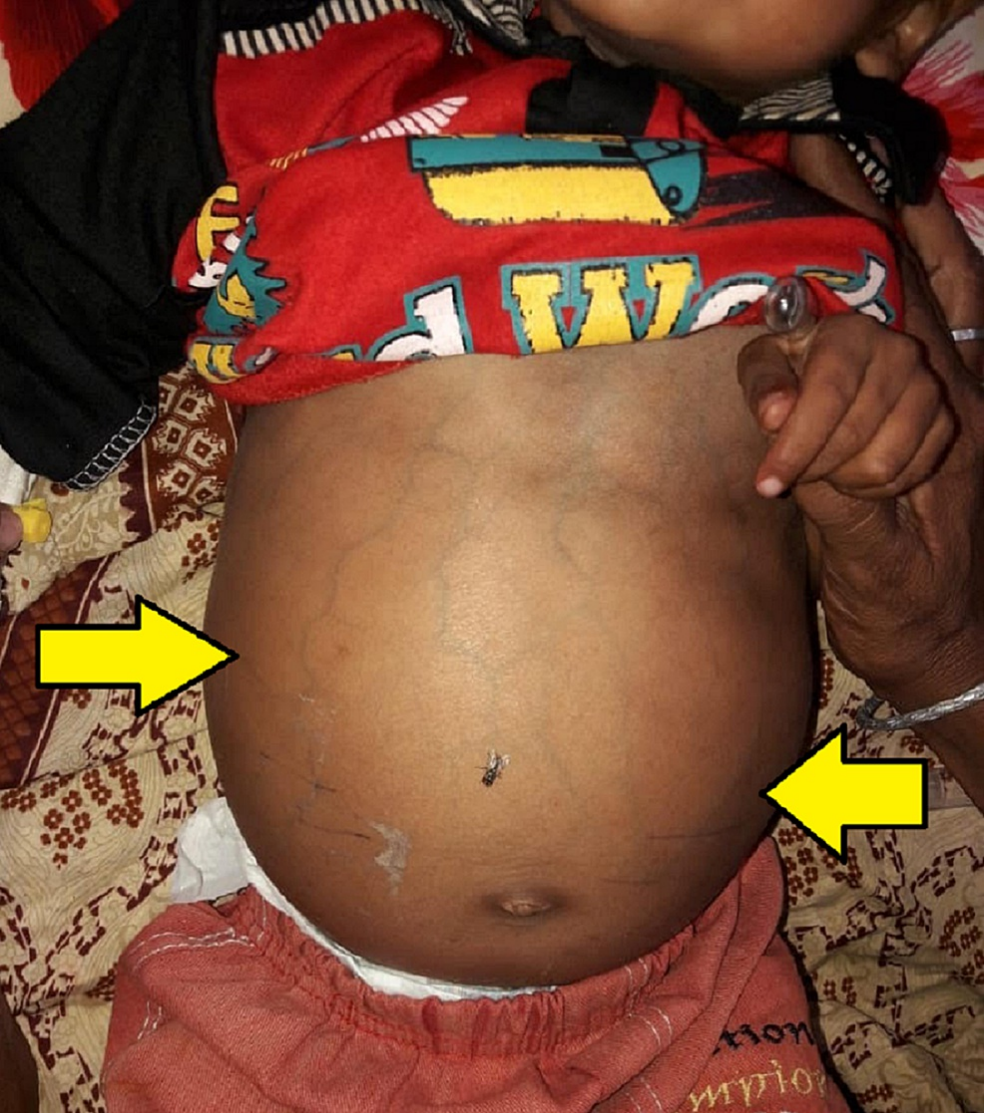
Marked ascites with increased abdominal girth (yellow arrows).

On general physical examination, he weighed 9.1 kgs and looked active, with no signs of distress or dysmorphic features. There was no history of sudden weight loss and the child had been underweight for his age. He had a fever of 101°F with a heart rate of 120 beats/min, blood pressure (BP) 102/62 mm/Hg, respiratory rate of 25 breaths/min, oxygen saturation of 98%, and capillary refill time (CRT) <2 secs. Anthropometric measurements showed a fronto-occipital circumference of 44 cm, length of 72 cm, and mid-upper arm circumference (MUAC) of 11.5 cm. The patient was severely edematous and anemic, with positive findings for palmar erythema. There were no signs of jaundice, cyanosis, dehydration, and clubbing. Abdominal examination revealed an overall hard, distended, and non-tender abdomen with audible gut sounds, shifting dullness, and a positive fluid thrill (Figure 1).

Within two weeks of admission, the abdominal girth progressively increased from 45 cm to 51 cm. Due to massive ascites, visceromegaly could not be appreciated on the physical exam. However, there were prominent tortuous veins visible on the abdomen. Examination of the cardiac and pulmonary systems was insignificant.

Abdominal tuberculosis, viral hepatitis, autoimmune hepatitis, Wilson’s disease, and chronic BCS were considered as differentials in diagnosis. Baseline investigations of the patient are reported in Table 1.

**Table 1:** Baseline investigations of the patient. Hb, hemoglobin; MCV, mean corpuscular volume; TLC, total leukocyte count; PLT, platelets; CRP, C reactive protein; BUN, blood urea; Cr, creatinine; PT, prothrombin time; APTT, activated partial thromboplastin time; INR, international normalized ratio; T Bil, total bilirubin; LDH, lactate dehydrogenase; ALT, alanine aminotransferase; AST, aspartate aminotransferase; and ALP, alkaline phosphatase.

Laboratory investigation	Normal value (unit)	Patient’s value (unit)	Comments
Hematological profile			
Hb	11-15.5 (g/dL)	9.4 (g/dL)	Low
MCV	71.3-84.0 (fL)	67.1 (fL)	Low
TLC	4-11 (×10^3^/μL)	17.8 (×10^3^/μL)	Raised
PLT	202-403 (×10^3^/μL)	233 (×10^3^/μL)	Normal
CRP	2-5 (mg/dL)	30 (mg/dL)	Raised
BUN	7-20 (mg/dL)	5 (mg/dL)	Low
Cr	0.3-0.7 (mg/dL)	0.3 (mg/dL)	Normal
PT	10.5 (seconds)	15.6 (seconds)	Raised
APTT	26-36 (seconds)	27.2 (seconds)	Normal
INR	≤1.10	1.5	Raised
Liver function profile			
T Bil	0.3-1.0 (mg/dL)	0.9 (mg/dL)	Normal
LDH	160-370 (U/L)	412 (U/L)	Raised
ALT	7-55 (U/L)	17 (U/L)	Normal
AST	8-60 (U/L)	21 (U/L)	Normal
ALP	142-335 (U/L)	238 (U/L)	Normal
Total protein	6.3-7.9 (g/dL)	4.5 (g/dL)	Low
Albumin	3.5-5.0 (g/dL)	2.7 (g/dL)	Low

Abdominal tuberculosis and viral hepatitis were ruled out as Mantoux tests, and viral markers were negative, respectively. Serological screening for autoimmune hepatitis was negative, and normal ceruloplasmin levels cleared suspicion of Wilson's disease. Screening for thrombophilic factors revealed borderline protein C value at 42% (normal: 40-92), while free protein S at 34.4% (normal: 67-140) and antithrombin levels at 59.1% (normal: 75-125) were low. The homocysteine level was 8.1 µmol/L (normal: 4.9-11.6 µmol/L). Tests for lupus anticoagulant and factor V Leiden yielded negative results. 

Blood, urine, stool culture, and sensitivity reports were negative with no bacterial growth. Ascitic fluid tap showed transudative findings (protein 2.1g/dL, 87% lymphocytes, polymorphonuclear leukocyte (PMN) 13% with a gram-negative stain and a serum-ascites albumin gradient (SAAG) ratio of 1.4), indicating portal hypertension.

**Figure 2:**
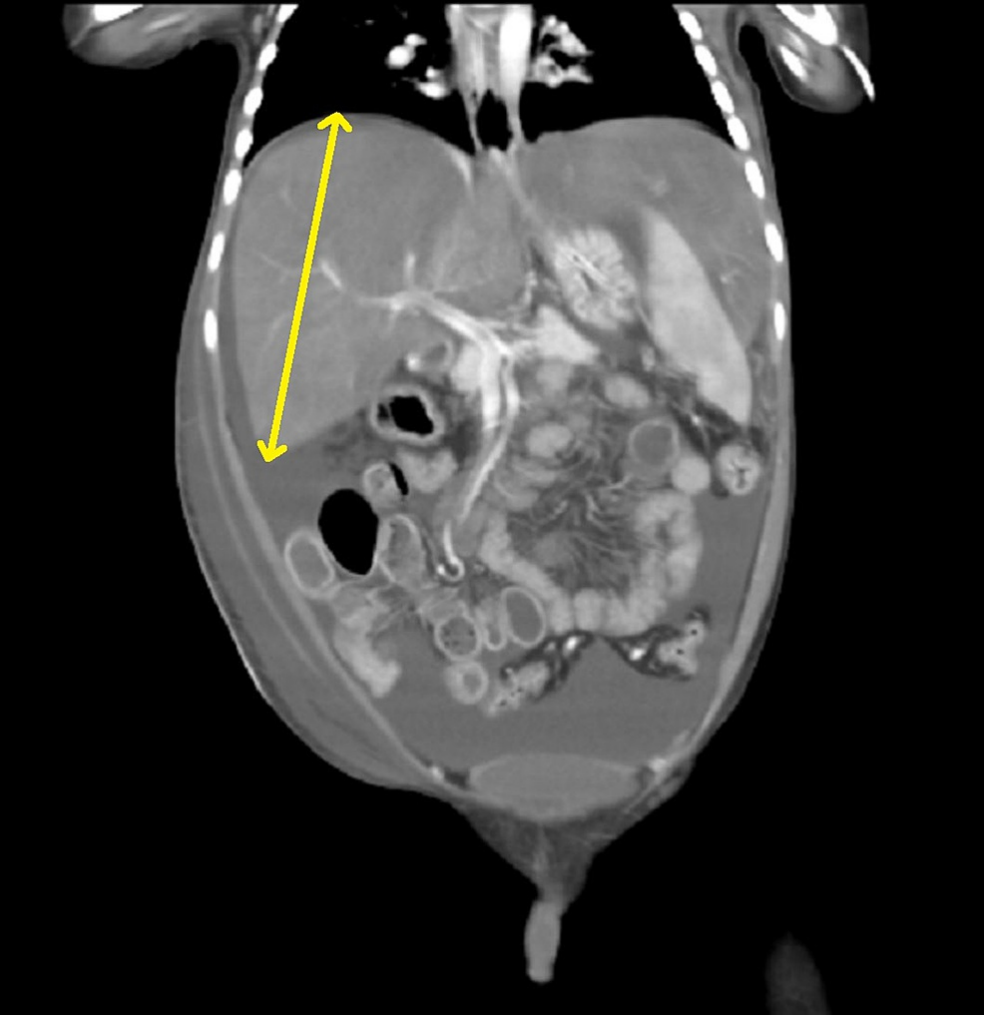
Coronal contrast triphasic CT scan showing massive hepatomegaly with homogenecity (yellow arrow). CT, computed tomography

**Figure 3:**
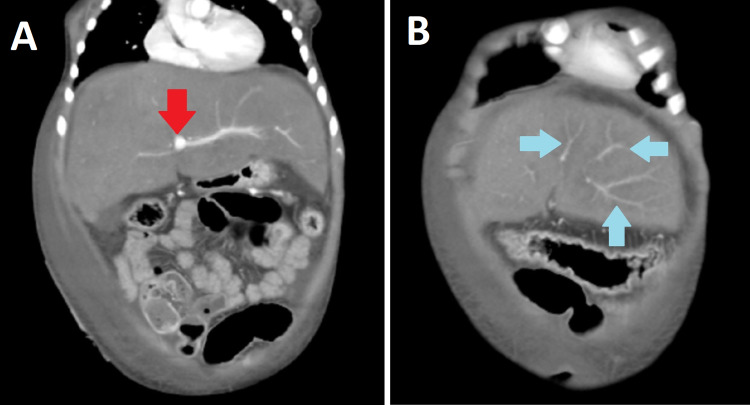
Axial contrast triphasic CT scan of abdomen. Image A shows occlusion of the portal vein (red arrow). Image B shows formation of collaterals in liver (blue arrows). CT, computed tomography

Fibroscan showed a Metavir score of 4, which was highly suggestive of cirrhosis. Abdominal ultrasound was performed and showed an enlarged liver span (Figure 2) with irregular margins and coarse echotexture. No focal mass and intrahepatic biliary duct dilatation could be appreciated. The spleen had a homogeneous parenchymal echo pattern with no focal mass and splenic vein dilatation. Triphasic CT scan findings showed a slit-like narrow caliber of IVC in the intrahepatic course (Figure 3). There was also a filling defect noted in the suprahepatic portion of IVC. Liver parenchyma showed altered parenchymal patchy enhancement on postcontrast images, with enlargement due to liver congestion with caudate lobe hypertrophy and gross ascites with no visualization of the hepatic vein. Endoscopy results of the upper gastrointestinal (GI) tract revealed grade 1 varices. The diagnosis of chronic BCS was made. 

On the day of admission, the patient was started on cefotaxime, amikacin, and metronidazole. However, the patient's condition did not improve even after 48 hours of administration. After one week, the antibiotics were changed to meropenem 200mg IV q8h, vancomycin 180mg IV q8h, and colomycin 455,000 I/U IV q8h along with spironolactone initiation, which was later replaced with furosemide. Upon gastroenterology and hematology consultation, anticoagulant therapy such as heparin was deemed unfavorable due to the chronic presentation. Radiological interventions such as percutaneous recanalization and transjugular intrahepatic shunts (TIPS) could not be performed due to a lack of expertise in the local setup. Ultimately the last resort for management was liver transplantation, for which the parents of the patient were counseled. However, due to financial constraints and the unavailability of donors, the procedure could not be performed. The patient was provided with symptomatic treatment, but failure to have a liver transplant led to the expiry of the patient a week later.

## Discussion

BCS is relatively uncommon in the pediatric age group, with maximum cases reported from the Indian subcontinent with male predominance [9]. The site of obstruction plays a significant role in determining the heterogeneity of the clinical and pathological manifestations amongst BCS patients. Additionally, the number of occluded hepatic veins and the speed of occlusion also significantly influence the course of pathophysiology [10]. 

Etiological classification of BCS can be done based on prothrombotic and non-thrombotic factors. The most common etiologies include hereditary thrombophilic disorders such as protein C and S, factor V Leiden, and antithrombin III (ATIII) deficiency. Acquired disorders, including myeloproliferative disorders, antiphospholipid syndrome, and paroxysmal nocturnal hemoglobinuria (PNH), are considered to be relatively less common [5,11,12]. Celiac disease, Behcet's disease, sarcoidosis, immunoallergic vasculitis, and granulomatosis are potential systemic prothrombotic causative factors of BCS. It may also occur due to obstruction of the hepatic vessels as a pathological consequence of pyogenic infections, hydatid disease, or benign or malignant tumors [13]. Comparably, the thrombophilia profile of our patient revealed antithrombin III deficiency and marginally decreased levels of protein C and S; it was classified as a classic case of BCS.

The clinical presentations of BCS vary widely due to the stage of disease, ranging from acute to chronic forms. On the grounds of acute BCS, the clinical presentation includes sudden onset hepatic insufficiency accompanied by ascites, abdominal pain, and renal failure [6]. However, the chronic presentation, the more common form of BCS, presents with a slower onset and fewer symptoms. It clinically manifests as cirrhosis and portal hypertension [7]. This was evident in our patient by a Metavir score of IV in fibroscan and a SAAG ratio of 1.4. Metavir score is a fibrosis staging system, and consists of five stages comprising of F0 (fibrosis absent), F1 (fibrosis with periportal expansion and absence of septa formation), F2 (fibrosis with periportal expansion and few septa formation), F3 (fibrosis with numerous septa formation and occasional nodules) and F4 (confirmed cirrhosis) [14]. Our case also presented with similar positive clinical findings for abdominal pain and ascites. Jaundice, ulcers in the lower extremities, and altered mental status are also observed in BCS but were not found in our patient [15].

The most crucial conclusion drawn for the diagnosis of pediatric BCS is through imaging techniques. Doppler US is considered the gold standard, owing to its diagnostic accuracy of 95% and its ability to demonstrate hepatic vein or IVC occlusion [16]. In our patient's report, doppler US portrayed hepatomegaly with coarse echotexture and gross ascites. Unfortunately, a Doppler scan of the portal and hepatic veins could not be performed due to patient's non-compliance. 

Other diagnostic methods such as triphasic CT and MRI are recommended in the literature when US results are ambiguous [17]. CT scans are usually done to further investigate liver morphology, parenchymal patterns, and vascular changes. The morphological changes generally seen in an abdominal CT scan of a BCS patient are caudate lobe hypertrophy and parenchymal enhancement patterns in the arterial phase [18].

The approach to BCS treatment follows a stepwise protocol as suggested in the literature [7,8]. After making a diagnosis of BCS, it is imperative to immediately start the patient on anticoagulation therapy while simultaneously ensuring that the underlying cause is treated. However, in cases of BCS that stem from prothrombotic states, anticoagulation therapy needs to be initiated under strict monitoring to avoid the risk of bleeding. In a study done by King's College Hospital, a BCS patient with an ATIII deficiency underwent a liver transplant but died three years later on warfarin therapy due to subarachnoid hemorrhage [9].

If the patient has severe signs and symptoms of BCS such as painful hepatomegaly, marked ascites, or progressive liver failure while being on anticoagulation therapy, the option of a transjugular intrahepatic portosystemic shunt (TIPS) can be considered as the next step in management protocols. If TIPS is unsuccessful, other interventional procedures such as percutaneous transluminal angioplasty (PTA) or percutaneous stent placement would be next on the consideration list. If all else fails, liver transplantation (LT) is advised as rescue therapy. [19]. Although there is literature supporting interventional radiological procedures for treating BCS in adults, minimal data is available for treating children [20]. Out of all the aforementioned interventional steps, LT is the most favorable in the pediatric age group, with lifelong anticoagulation therapy a second option.

## Conclusions

BCS is a rare cause of liver cirrhosis in the pediatric population. It may present with symptoms such as poor feeding, diarrhea, abdominal distension, and pain. The symptoms may have acute or chronic history, and due to vague presenting signs and symptoms, diagnosis is often delayed. It may also be misdiagnosed as infection; therefore, physicians must keep BCS as a differential when a child presents with the aforementioned symptoms. Delayed diagnosis and controversial treatment guidelines of BCS in children contribute to increased mortality rates. Early intervention and management of BCS are crucial due to the poor prognosis of the disease. This case report highlights the pivotal role that timely diagnosis and stepwise management protocols play in improving the outcome and course of the disease.
